# A measurement tool to capture sexual harassment among women in Tanzania: results from cognitive testing

**DOI:** 10.1080/16549716.2026.2653923

**Published:** 2026-04-29

**Authors:** Joyce Wamoyi, Meghna Ranganathan, Heidi Stöckl

**Affiliations:** a Department of Sexual and Reproductive Health, National Institute for Medical Research, Mwanza, Tanzania; b Department of Global Health and Development, London School of Hygiene and Tropical Medicine, London, UK; c Institute for Medical Information Processing, Biometry and Epidemiology, Ludwig-Maximilians-Universität München (LMU Munich), Munich, Germany

**Keywords:** Measurement, sexual harassment, women, Tanzania, cognitive testing

## Abstract

**Background:**

Sexual harassment is a serious public health problem, yet there is a lack of clarity on its measurement.

**Objective:**

We set out to develop a measurement tool to capture sexual harassment in the different spheres of women’s everyday lives.

**Methods:**

We conducted 60 cognitive interviews with women in two iterations. In each iteration, we interviewed 10 young women aged 15–24 years, sampled from two public secondary schools; 20 women aged 25 years and above sampled from public spaces and workplaces. Iteration one explored five key questions involving concepts on unwelcome or unwanted touching, signals, and/or words or comments of sexual nature from the boy/man and a direct question on ‘ever experiencing sexual harassment’. Only three questions were explored in iteration two with the additional layer of context.

**Results:**

Majority of the participants were able to grasp the key concepts. However, they were likely to respond ‘yes’ to questions that were less descriptive and that included additional information on context where harassment happened. A very explicit question, ‘ever experienced sexual harassment’ appeared stigmatising and yielded fewer affirmative responses from the same individuals. Older women understood the questions earlier than adolescent girls who were more likely to ask for clarification and a repeat of the questions.

**Conclusion:**

An optimal question for measuring sexual harassment must include the context for the behaviour and avoid stigmatising participants. We propose a comprehensive question that includes all forms of harassment and that can be adapted for measuring the practice in different populations and contexts.

## Paper context

- **Main findings:** An optimal question for measuring sexual harassment must include context for the behaviour, should not be stigmatising, and should be adaptive to the setting and population under investigation.
- **Added knowledge:** An optimal question for measuring sexual harassment includes context for the behaviour, is less stigmatising, and is adaptive to other settings and populations.
- **Global health impact for policy and action:** Our study findings have implications on how sexual harassment is measured among girls and women in diverse settings and for comparability of data on its magnitude.

## Background

Sexual harassment is a global public health problem and negatively affects individuals‘ mental health, wellbeing, aspirations, and their behaviour [1–4]. The World Health Organisation (WHO) defines it as ‘any unwelcome sexual advance, unwelcome request for sexual favour, verbal or physical conduct or gesture of a sexual nature, or any other behaviour of a sexual nature that might reasonably be expected or be perceived to cause offence, humiliation or intimidation to the person’ [5]. While everyone can be a victim or survivor of sexual harassment, evidence shows that women are significantly affected [6]. The ground-breaking anti-sexual assault and harassment movements, Time’s Up and #MeToo, elevated global awareness of the offending actions that women encounter in their daily personal and working lives. Although we have a better understanding of how sexual harassment is conceptualised and perceived in low- and middle-income countries (LMICs) [7], with specific country-level research in Tanzania [8], there is a lack of clarity on how best to measure sexual harassment quantitively to determine its true prevalence [9].

Through the exploration of narratives on people’s experiences of sexual harassment, we now have a good understanding of the definition and multi-dimensional nature of sexual harassment with the key feature being ‘consent’ and ‘power’ [7,8]. In a systematic review conducted by Ranganathan et al. [9], it was observed that many studies used an unclear definition of sexual harassment and did not deploy a validated measurement tool making it difficult to compare findings across studies, leading to either an underestimation or potential overestimation of the issue. The studies reviewed either used a direct question or a series of behavioural questions to elicit information on acts considered offensive or defined as sexual harassment. Hence, there is a lack of clarity on the prevalence and measurement of sexual harassment in LMICs, making it challenging to compare prevalence results from different settings. Measurement tools for sexual harassment have mainly been developed and tested in high-income countries – either with uncertain relevance for the women in the Global South or that they were too long to include in routine surveys. Further, tools used by the International Labour Organisation (ILO) and the WHO to measure abuses are applicable only for specific spheres of life, e.g. work or education. Our study offers a first step towards understanding how these abuses can be measured in Tanzania across a variety of spheres. The WHO multi-country study on violence against women indicates that Tanzania is one of the countries with the highest prevalence of non-partner sexual violence, with 11.5% in Mbeya and 9.4% in Dar es Salaam [10]. The 2016 National Action Plan to End Violence against Women and Children in Tanzania explicitly mentions the need to reduce sexual harassment in the workplace and to end all other forms of sexual abuse [11]. The aim of the proposed study is to develop a measurement tool to capture sexual harassment in different spheres of women’s everyday lives. The tool could improve the measurement of sexual harassment in low resource settings.

## Methods

We conducted cognitive interviews with girls in school, women in public places, and women in workplaces in Mwanza city in Tanzania. Our methodological approach was informed by Miller’s [12] guide to cognitive interviewing technique. We chose cognitive interviewing because of its iterative process, wherein a first set of ‘participants’ identify major problems with the wording and structure of a research question, followed by efforts to improve on these issues. The research team then re-tests the revised question [12], with a second iteration of cognitive interviews. Through this process, the internal validity of survey questions is assessed and it ensures that the resulting measures reflect the phenomenon/behaviour of interest [12], which in our case is the measurement of sexual harassment.

### Study population

The study population included adolescent girls aged 15–18 years in secondary schools; young women aged 18–24 out of school; and adult women aged 25 and above. We chose varied study populations for this study because the understanding and experience of sexual harassment may differ between different places, age groups, and sex [7,8]. A total of 60 women participated in cognitive interviews (30 in iteration one, while the remaining in iteration 2). Those sampled had not participated in any of the other previous research activities related to this study.

In circumstances where there have been attempts to measure sexual harassment, the questions that have been used to measure prevalence have varied significantly [9]. This may have been because the definition of sexual harassment varied in the different studies, or the measures did not capture the practice of interest or were conflating it with general sexual violence. Therefore, we conducted cognitive interviews with female participants from groups identified as likely to experience sexual harassment to develop a measure to capture the prevalence of the practice in LMIC settings, such as Tanzania.

The development of research questions for the measurement tool was based on findings from qualitative and quantitative systematic reviews on the conceptualisation and measurement of sexual harassment [9] and qualitative in-depth studies in Tanzania to develop our conceptual framework for sexual harassment based on individuals’ perceptions and reported experiences of sexual harassment [7,8]. This ensured that the development of our research questions encompassed critical aspects emerging of the above studies: (a) Consent (any unwelcome sexual advance, unwelcome request for sexual favour, verbal or physical conduct or gesture of a sexual nature, or any other behaviour of a sexual nature that might reasonably be expected or be perceived to cause offence) and (b) gender power (social, economic, and political dynamics that shape the distribution of influence, decision-making, and resources between genders). By testing and refining the measurement tool through two iterations, we ensured that the resulting measure reflects the phenomenon and behaviour of sexual harassment and thereby assessed its internal and face validity of survey questions.

In total, the cognitive interviews were conducted in two iterations among (a) 10 young women aged 15–24 were sampled from two public learning institutions (secondary schools); (b) 10 women aged 25 and above sampled from public spaces; (c) 10 sampled from workplaces (government offices). Participants for the 2nd iteration were recruited in the same numbers and distribution from the study, age, and setting as in the 1st iteration, but different study participants from these groups were recruited.

### Participant recruitment

We used purposive and snowball sampling to recruit participants for cognitive interviews as snowball sampling allows participants to select peers with similar characteristics to them.

### Recruitment of participants in public places (market)

After obtaining permission from the ward level authorities, the research assistants visited two public marketplaces in Mwanza town. Sampling of study participants was conducted with the help of the ward level authorities. Initially, we contacted three women who work at the market. The researcher explained the purpose of the study and sought their permission to participate in the study and in helping with recruitment of others in the public places where they spend time. As the researchers got to know more participants, purposive sampling was employed to have a total of 12 participants. The above procedure was repeated in iteration 2.

### Recruitment of participants from educational institutions (secondary schools)

Approximately four public secondary schools in the two districts were approached to participate in the study. Initial permission to visit the schools was sought from the district education officers. Additional permission was sought from the ward education office. On arrival at the selected secondary schools, introductions were done with the school head teachers and permission sought to speak with girls aged 15–18 years. The teachers introduced three girls to the researchers with a range of characteristics such as age (e.g. those aged 15–16 versus older ones 17–19) and level of schooling (e.g. A-level versus O-level). These initial contacts were asked to introduce the researchers to their network of friends who were either in the same class as them or a different class but within the same school. The researcher met with the contacts, explained the study objectives, and asked those willing to participate in the study to an interview. In addition to child assent, parental/guardian consent was sought for all students under the age of 18 years. The above procedure was repeated in iteration 2.

### Recruitment of participants in workplaces

Recruitment of participants for workplaces took place at the Mwanza regional government offices and the Mwanza municipal council. After seeking permission from the regional authorities, the researcher sent a request letter introducing themselves and the study and sought appointments with the directors at the municipal council and regional offices. The researcher sought permission for staff of different cadres to participate in the study. The invites for the participants were done with the help of the human resource managers who send out invitations to two people from each cadre/department to participate in the study.

### Procedure for cognitive interviews

The cognitive interviews involved a one-on-one in-depth and highly focused interview. After collecting basic socio-demographic information such as age and education level, we asked the participant to read or listen to a question that we intend to use in surveys to measure sexual harassment. We then used the techniques of ‘verbal probing’ to dissect the response given by the participant. This verbal probing technique allowed the interviewer to use additional questions to explore the basis for the response further.

Female interviewers conducted the interviews in a private location selected in collaboration with the study participants. The interviewers were not members of the research team. Upon arrival at the location of the interview, the interviewer welcomed the participant and explained the study in general, introduced the specific topic of discussion, and explained reasons for conducting the study.

Due to the sensitivity of this topic and the cultural tradition in the study context, participants were interviewed by female interviewers. Cognitive interviews took approximately one and half hours and were audio recorded in addition to interviewers taking notes. The interviewers were trained social scientists with a Bachelor’s or Master’s degree and with experience conducting research on sensitive topics.

Rapport was established with all participants, and prior to the interview, participants were offered lunch or snacks. The interviews followed the topic guide. In both iterations of data collection, the topic guide consisted of questions that assessed participants’ understanding of the question asked what they would have responded to the question and why, whether they needed the question to be repeated for them to respond, and how they repeated the question in their own words.

### Ethics and informed consent

Our study adheres to the principles of Declaration of Helsinki. The study received ethical approval from Tanzania National research ethics committee (NIMR/HQ/R.8a/Vol.IX/4254) and LSHTM (Ref: 16,116).

Informed written consents were taken from all participants prior to participation in the study because all were able to read and write. For participants below the age of 18 years, parental informed written consents were obtained in addition to adolescent written assent.

### Analysis

The cognitive interview content analysis began with the daily interview debriefs with all the interviewers. The summaries were prepared by the individual interviewers in Excel sheets and discussed among the investigators. Analysis continued until we achieved data saturation [13]. The first iteration had a total of five key questions, while the second one had three questions that had been refined from the first iteration analysis (see Table 1). The groups were analysed separately.

**Table 1.** Findings from iteration 1.

Questions asked	Schools	Workplaces	Public places
Q.301. Did you experience unwelcome/unwanted touching, signals and or words/comments of sexual nature from the boy/man? repeated question? Yes/No	Although all adolescents responded ‘yes’ to the question they asked the interviewer to repeat the question for clarification	9/10 asked the interviewer to repeat the question and suggested alternative wording	8/10 participants clearly understood question. Only two asked for the question to be repeated
Q.302. Did a man/boy touch you, signal you or speak/comment to you in a sexual manner you consider unacceptable or displeasing to you? YES/NO	7/10 adolescents asked for the question to be repeated for clarification	3/10 understood the question and paraphrased it in their own words; 2/10 asked the researcher to repeat the question.	3/7 who responded to this question asked for it to be repeated for clarification
Q.401. Have you, personally, EVER experienced SEXUAL HARASSMENT?	6/10 adolescents asked for the question to be repeated	6/10 understood the question and did not want it repeated. They reported that peers answer ‘NO’ as it is too direct.	5/10 participants asked for clarification. They reported the question was too direct and uncomfortable to answer.
Q.402. Have you, personally, EVER experienced SEXUAL harassment such as unwanted/unwelcome sexual touching, comments, pictures, emails, or sexual requests?	Adolescents reported that the question is confusing	6/10 reported the question is clear. However, the examples could be confusing if combined	All found the question confusing and believed it could lead to people answering ‘NO’
500. Have you ever experienced unwanted sexual advances or behaviours that made you feel uncomfortable or afraid by someone else than your partner, for example at school, work or public transport?	3/10 reported yes to this question. The rest did not answer	Only 2/10 responded, yes? to this question and tried to paraphrase it	All understood the question but believed it could lead to people answering ‘NO’ as it is too direct

## Results

We explored five key questions in iteration one. We summarise the findings in Table 1.

Based on the lessons learnt in iteration one, we only moved ahead with three questions in iteration 2. The questions were revised for clarity as presented in Table 2.

**Table 2.** Findings from iteration 2.

Questions asked	Schools	Workplaces	Public places
301. Has a boy/man ever touched you, signalled to you or made comments of a sexual nature that were unwanted by you?	The question was clear for 8/10 girls, only 2/10 asked for repeat/elaboration. 3/10 reported the question was not easy to answer.	This question was easily understood by most women in the workplace	This question was easily understood by women in public places
302. Has a boy/man ever touched you, signalled to you or made comments of a sexual nature that were unwanted by you? For example, at school, workplace or public places? [CONTEXT ADDED]	All girls reported that this question was easy to understand as it gives context, and that majority of girls would answer ‘yes’	All reported that this question is easy to answer as it is specific compared to Q301.	Question was easy to answer and better than Q301 due to addition of context
500. Have you ever experienced unwanted sexual advances or behaviours that made you feel uncomfortable or afraid by someone else than your partner, for example at school, work or public transport?	6/10 asked for the Q500 to be repeated for clarity but also reported they felt uncomfortable answering the question.	This direct question is clear but not easy to answer. Women will be ashamed to respond ‘YES’ to such a direct question. Hence Q301 & 302 are better worded to generate honest responses.	4/10 asked for the question to be repeated This was a direct question, but they thought Q301 and Q302 would elicit more honest responses compared to Q500

Based on the lessons learnt in iteration one, we only moved ahead with three questions in iteration 2. The questions were revised for clarity as presented in Table 2.

Most of the participants were able to grasp the key questions in both iterations. A few were able to paraphrase the question in their own words. Participants reported that other/peers would not answer honestly to questions 401 and 402 in iteration one due to the explicit nature of the questions. Many were not able to respond to the explicit question 501 themselves.

Although a majority of adolescent girls reported that they had understood Q 301 (Iteration 1), when asked to explain what the question meant their explanations revealed different meanings with majority saying they understood the question to mean ‘being forced to have sex’. Others reported they thought the question was about sexual relationships.

The wording of sexual harassment questions is key for valid measurement of the behaviour. We noted that simple wording such as ‘touched inappropriately’ may not be culturally appropriate resulting in unnecessary stigmatisation of the participant. The inclusion of the wording ‘experience of touching’ was also problematic as it connoted that the girl or woman was used to touching as an indication of immoral behaviour. We also observed that a direct question like ‘Did you experience sexual harassment’ resulted in the under reporting of the experiences of the behaviour. Although this question was clear and well understood, it was stigmatising as it related to immorality with most participants reporting that they and their peers would respond ‘No’ when asked such a question.

In iteration 2, question 302 was easily understood with all adolescents reporting that the addition of context to it had made it clearer. Many adolescents found it difficult to answer Q. 500. This could be due to its direct nature that caused discomfort among participants. When asked to compare Q500 and 301, all adolescent girls reported that even without context, Q301 was easier to answer compared to Q500.

Adults from workplaces and public places seemed to easily understand the three questions in iteration two compared to adolescent girls. Overall, when asked to compare three questions question 301, question 302, and question 500 based on the levels of ease or comfort in being able to answer and one that would elicit a honest response, all the three categories of women felt that question 302 was easily understood and that it would encourage women to give more honest responses compared to questions 301 and question 500. Specifically, many reported that question 500 was too direct and caused feelings of shame when responding to it and thus discouraged honest reporting.

## Discussion

Although emerging evidence points to sexual harassment as being common, the magnitude of the practice has not been well measured across settings and groups in Tanzania or globally. We set out to develop a measure for measuring sexual harassment in multiple populations and diverse contexts in Tanzania. Based on our cognitive interviews with adolescent girls and adult women at schools, workplaces, and public places, we recommend question 302 *‘Has a boy/man ever touched you, signalled to you or made comments of a sexual nature that were unwanted by you, for example, at school, workplace or public places?*’ as the best to capture women’s multiple experiences of sexual harassment quantitatively, if only a single question is possible.

The word *unwanted* ensures that the overlap that exists between behaviours that constitute sexual harassment and seduction in the Tanzanian setting is clear and captures the crucial components consent and gender power that have been previously established in local and international work on the definition and conceptualisation of sexual harassment [7–9]. Acts of sexual harassment are often conflated with seduction and courtship and thus making it difficult to differentiate the two behaviours [7,8]. This confusion coupled with sexual norms on what is acceptable versus not in public has often resulted in under reporting.

As girls and women often suffer from stigma and shame upon experiencing sexual harassment, the behaviour is often not reported or discussed. In our study, as in previous studies, we observed that women were not comfortable reporting explicit sexual behaviours, including acts of sexual violence or sexual harassment [14–16]. Most girls and women in the cognitive interviews who reported experiencing sexual harassment therefore reported that their peers and them were more likely to report yes to questions asking for this if the question was less direct, outlining acts but not using terms like sexual harassment. The addition of context regarding the settings in which sexual harassing acts might have occurred added clarity and recall among participants as it reminded them about specific incidents and made them consider more than one setting. In our case, we included schools, workplace, and public places but this question could also be adapted to include sexual harassment at home and in other contexts such as humanitarian settings where this behaviour is also common [17]. In our previous qualitative work, participants also reported sexual harassing behaviours by neighbours, extended family members, or social networks [18]. Hence, we recommend that the question should also include additional settings beyond the traditional ones, depending on the specific contexts on where and for which population the interviews are being conducted. As we have not explored sexual harassment by relatives at home, we are therefore not sure if its nature differs as the perpetrators are likely to be related to the girl or woman compared to perpetrators who are total strangers outside the home. However, we recommend future work on this for potential inclusion. We recommend that the questions are piloted out in different cultural contexts and adapted to suit the study population.

One of the major limitations of our recommended question relates to the lack of probes on the perpetrator of the sexually harassing behaviour. Perpetrator related questions can be added, after prior testing after the responded answered positively or within a detailed question. We have not tested this in this study as we prioritised a single question that could assist in estimating an overall prevalence of the occurrence of sexual harassment regardless of perpetrator relationships. More detailed studies on the phenomena would require this question though; hence, we recommend further work on it.

Another limitation of the recommended measurement question is that it may not work for the measurement of sexual harassment in boys and men or female on female sexual harassment. As noted in other studies, men and boys are also likely to experience sexual harassment but may struggle to report the practice for fear of stigma [19]. In fact, in our qualitative studies, men rarely reported it [7,8], although we explicitly inquired about it. Finally, participants may in some instances have answered questions in a way they believed was more acceptable, rather than accurately reporting their cognitive process and thus introduced social desirability bias.

### Data credibility

We attempted to ensure the credibility of our findings via several approaches. First, we collected data from diverse populations of women (public places, workplaces, and schools). Second, we engaged five researchers to ensure reliability in the analysis and interpretation of data. The data collection team was familiar with the study context and had previously worked in these contexts. Although researcher familiarity with context was advantageous, it may have resulted in a social desirability bias.

### Reflexivity

The study team was composed of individuals with expertise in sexual violence research in sub-Saharan Africa. The first author was involved in the first phase of the study exploring conceptualisation of sexual harassment in Tanzania. While this was beneficial in many ways, it could have unintentionally resulted to bias in how she approached the analysis and interpretation of the data.

## Conclusions

A lack of consent and gendered power are key defining features of the practice of sexual harassment and are reflected in our final suggested question, ‘*Has a boy/man ever touched you, signalled to you or made comments of a sexual nature that were unwanted by you, for example, at school, workplace or public places*?’. The suggested question is comprehensive as it includes all forms of harassment (e.g. verbal, physical, and emotional) and could be adapted for measuring the practice in different study populations and contexts.

We recommend the following key considerations that constitute a good question to measure sexual harassment: (1) Question must combine different forms of sexual harassment in one, especially if the question is embedded in a study with less room for multiple/individual questions on different forms of sexual harassment; (2) Measurement must ensure that the question captures context as this was critical for recall and reporting; (3) Question should not be explicit in its wording using words such as ‘sexual harassment’ as this could lead to stigmatisation of the behaviour resulting in the underreporting of the behaviour.

Improved measures of shameful, but seemingly widespread sexual behaviours, such as sexual harassment are important for researchers, programmers, and policy makers to get accurate estimates of the prevalence and association with undesirable health outcomes among women. A measure that can capture the practice of sexual harassment accurately across populations and contexts is critical for designing and targeting of programmes to reduce the everyday abuse of women and girls. Our measure has the potential to be used in national surveys yet requires piloting in different populations and settings to test their reliability and validity.

## Supplementary Material

COREQ Supplementary.doc

## Data Availability

Data would be available upon reasonable request from the authors.
