# Supplementary material for: A measurement tool to capture sexual harassment among women in Tanzania: results from cognitive testing
Source: Glob Health Action. 2026 Apr 29;19(1):2653923. doi: 10.1080/16549716.2026.2653923 (PMC13130241; doi:10.1080/16549716.2026.2653923)
Supplement: COREQ Supplementary.doc [file ZGHA_A_2653923_SM7847.doc]

**Manuscript: A measurement tool to capture sexual harassment among women in Tanzania: results from cognitive testing**

**Consolidated criteria for reporting qualitative studies (COREQ): 32-item checklist**

| **No. Item** | **Guide questions/description** | **Reported on Page #** |
| --- | --- | --- |
| **Domain 1: Research team and reﬂexivity** |  |  |
| *Personal Characteristics* |  |  |
| 1. Interviewer/facilitator | None of the authors conducted the interviews. However, data collection was done by a team of well-trained graduate researchers. | Page 7 |
| 2. Credentials | 2 data collectors had a masters while one had a bachelors degree.  The investigators and lead analysts had PhD | Page 1 and 8 |
| 3. Occupation | Researchers | Page 1 and 8 |
| 4. Gender | Female and males | Page 1 and 8 |
| 5. Experience and training | Trained in research ethics and data collection skills | Page 1 and 8 |
| *Relationship with participants* |  |  |
| 6. Relationship established | Yes (rapport established with all participants) | Page 8  . |
| 7. Participant knowledge of the interviewer | Yes. There was researcher introduction of themselves and the reasons for doing the research. | Page 7 and 8 |
| 8. Interviewer characteristics | Reasons and interests in the research topic | Page 7 and 8 |

| **Domain 2: study design** |  |  |
| --- | --- | --- |
| *Theoretical framework* |  |  |
| 9. Methodological orientation and Theory | Content analysis | Page 9 |
| *Participant selection* |  |  |
| 10. Sampling | Purposive and snowball sampling | Page 6 and 9 |
| 11. Method of approach | Face-to-face | Page 5 to 9 |
| 12. Sample size | 60 | Page 2 and 5 |
| 13. Non-participation | None | N/A |
| *Setting* |  |  |
| 14. Setting of data collection | Schools, workplace, community halls, homes | Page 6 to 9  . |
| 15. Presence of non-participants | No | N/A |
| 16. Description of sample | Adolescent girls aged 15-18 years in secondary schools; young women aged 18-24 out of school; and adult women aged 25 and above | Page 5 to 9 |
| *Data collection* |  |  |
| 17. Interview guide | Yes |  |
| 18. Repeat interviews | No | No |
| 19. Audio/visual recording | Yes | Page 8 |
| 20. Field notes | Yes | Page 8 |
| 21. Duration | 90 minutes | Page 8 |
| 22. Data saturation | Yes | Page 9 |
| 23. Transcripts returned | Yes, validation was done in the second round of data collection, iteration 2 | Page 6 to 8 |
| **Domain 3: analysis and ﬁndings** |  |  |
| *Data analysis* |  |  |
| 24. Number of data coders | 3 | Page 8 |
| 25. Description of the coding tree | Cognitive interview utilizes summaries and debriefs as the process of analysis. Yes, summaries provided in Tabel 1 and 2 | Page 8 and 9 |
| 26. Derivation of themes | Yes, the refinement of measurement questions was the goal and hence, the analysis centered on the understanding of the questions. | Page 9 to 12 |
| 27. Software | No it was not necessary given the focus of the cognitive interview approach | Page 9 |
| 28. Participant checking | Yes, The cognitive approach involves iterations with participants and discussion of the findings at each stage | Page 7 to 9 |
| *Reporting* |  |  |
| 29. Quotations presented | Yes, presented in a different format in line with cognitive interview approach. | Page 9 to 13 |
| 30. Data and ﬁndings consistent | Yes | Page 9 to 13 |
| 31. Clarity of major themes | Yes | From page 9 to 13 |
| 32. Clarity of minor themes | Yes | Discussion of the measurement. From page 9 to 13 |
